# Quantitative Computed Tomography Features for Predicting Tumor Recurrence in Patients with Surgically Resected Adenocarcinoma of the Lung

**DOI:** 10.1371/journal.pone.0167955

**Published:** 2017-01-09

**Authors:** Hyun Jung Koo, Yu Sub Sung, Woo Hyun Shim, Hai Xu, Chang-Min Choi, Hyeong Ryul Kim, Jung Bok Lee, Mi Young Kim

**Affiliations:** 1 Department of Radiology and Research Institute of Radiology, Asan Medical Center, Seoul, Korea; 2 Department of Radiology, The First Affiliated Hospital of Nanjing Medical University, Nanjing, Jiangsu Province, China; 3 Department of Oncology, Asan Medical Center, Seoul, Korea; 4 Pulmonary and Critical Care Medicine, Asan Medical Center, Seoul, Korea; 5 Thoracic and Cardiovascular Surgery, Asan Medical Center, Seoul, Korea; 6 Clinical Epidemiology and Biostatistics, University of Ulsan College of Medicine, Asan Medical Center, Seoul, Korea; Stanford University Medical Center, UNITED STATES

## Abstract

**Purpose:**

The purpose of this study was to determine if preoperative quantitative computed tomography (CT) features including texture and histogram analysis measurements are associated with tumor recurrence in patients with surgically resected adenocarcinoma of the lung.

**Methods:**

The study included 194 patients with surgically resected lung adenocarcinoma who underwent preoperative CT between January 2013 and December 2013. Quantitative CT feature analysis of the lung adenocarcinomas were performed using in-house software based on plug-in package for ImageJ. Ten quantitative features demonstrating the tumor size, attenuation, shape and texture were extracted. The CT parameters obtained from 1-mm and 5-mm data were compared using intraclass correlation coefficients. Univariate and multivariable logistic regression methods were used to investigate the association between tumor recurrence and preoperative CT findings.

**Results:**

The 1-mm and 5-mm data were highly correlated in terms of diameter, perimeter, area, mean attenuation and entropy. Circularity and aspect ratio were moderately correlated. However, skewness and kurtosis were poorly correlated. Multivariable logistic regression analysis revealed that area (odds ratio [OR], 1.002 for each 1-mm^2^ increase; *P* = 0.003) and mean attenuation (OR, 1.005 for each 1.0-Hounsfield unit increase; *P* = 0.022) were independently associated with recurrence. The receiver operating curves using these two independent predictive factors showed high diagnostic performance in predicting recurrence (C-index = 0.81, respectively).

**Conclusion:**

Tumor area and mean attenuation are independently associated with recurrence in patients with surgically resected adenocarcinoma of the lung.

## Introduction

Small asymptomatic lung cancers are usually detected during computed tomography (CT) screening [1]. With the increase in detection of early cancers, the classification of lung adenocarcinoma was changed by the International Association for the Study of Lung Cancer, American Thoracic Society, and European Respiratory Society [2], and preinvasive lesions and minimally invasive adenocarcinoma were introduced. Ground-glass attenuation has been considered as an important prognostic factor for tumor recurrence [3, 4] and corresponds to a lepidic growth pattern of the tumor cells [2]. In addition, visceral pleural invasion and lymphovascular invasion have been suggested as criteria for predicting patients’ survival [5, 6].

In terms of radiology, there have been recent attempts to establish the radiologic correlates of the pathologic classification of lung adenocarcinomas in order to predict disease-free survival and outcomes [7, 8]. A systematic method for differentiating recurrence from non- recurrence of adenocarcinoma of the lung is important given that there is concern regarding the use of adjuvant therapy versus watchful follow-up after surgical resection. If there is high risk of recurrence, scrutinize follow-up schedule could be planned after surgery. To provide objective quantitative values rather than visual assessment, texture analysis of tumors has been suggested as a potential source of prognostic biomarkers [9–15]. Entropy, skewness, and mean attenuation were analyzed to identify radiologic independent prognostic factor for patients with non-small cell lung cancer [16, 17]. However, there are limited studies to investigate the value of CT texture analysis compared with the clinical and other radiologic prognostic factors to predict tumor recurrence in surgically resected lung adenocarcinoma [11, 18]. If quantitative CT features including histogram analysis could be used to predict tumor recurrence in a clinical setting, this would help in making treatment decisions and in follow-up plans to improve outcome in surgically resected lung adenocarcinoma. The purpose of the study was to retrospectively perform quantitative CT analysis of lung adenocarcinoma to assess their association with tumor recurrence in patients with resectable stage I and II lung adenocarcinoma treated by surgery.

## Materials and Methods

The institutional review board of our hospital approved this retrospective study (Approval 2015–0725) and the requirement for informed consent was waved.

### Study Population

**Fig 1:**
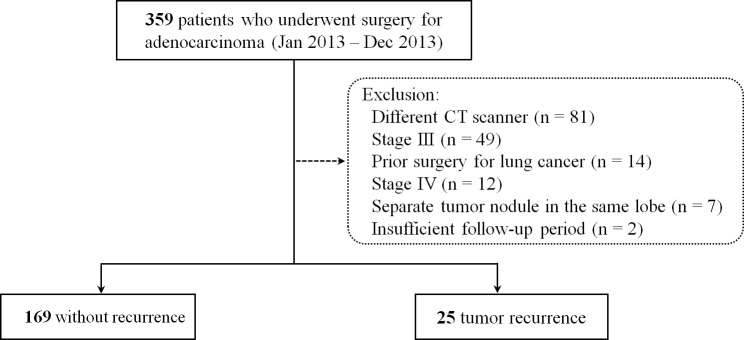
Flow diagram of the study patients.

According to the lung cancer registry at our institution, 359 patients underwent complete surgical resection (R0) between January 2013 and December 2013. Inclusion criteria were (a) no separate tumor nodules in the same lobe; (b) follow-up exceeding 6 months after tumor resection; and (c) standard preoperative contrast-enhanced CT obtained with one dedicated CT scanner, with both 1-mm and 5-mm thickness images. To perform a per-patient basis analysis of the tumor, patients who had separate tumor nodules were excluded. After excluding patients with CT obtained with a different scanner (n = 81), prior surgery for lung cancer (n = 14), stage III or IV (n = 61), separate tumor nodules (n = 7), and insufficient follow-up period (n = 2), 194 patients (81 males and 113 females) with pathologic stage I-II lung adenocarcinoma were selected (Fig 1). The final pathologic stages were graded based on the 7^th^ edition of the International Association for the Study of Lung Cancer [19, 20]. In the present work, we analyzed quantitative CT features using a dedicated CT scanner to avoid potential variability from the use of different CT scanning parameters. Standardized preoperative staging work-up using chest CT, bronchoscopy or CT/fluoroscopic guided biopsy, and PET/CT were performed in all patients. Tumor recurrence, quantitative CT characteristics, maximum standardized uptake value on PET/CT, and pathologic data were carefully reviewed. In addition, clinical, radiologic, and pathologic findings were analyzed and compared in patients with and without tumor recurrence.

### Clinical Data

Clinical data were obtained from the electronic medical records, including patient demographics, smoking status, surgical T stage, N stage, and date of tumor recurrence. Pathologic data gathered comprised % lepidic growth pattern, presence of lymphovascular invasion and of epidermal growth factor receptor, and predominant patterns such as micro papillary or solid components.

### CT Examinations

Preoperative CT imaging was performed within 2 weeks prior to the surgery. CT scans were performed with a 16-detector row scanner using a SOMATOM Sensation 16 (Siemens Medical Solutions, Erlangen, Germany). The following scan parameters were used: 120 kV; 100 effective mA with dose modulation; reconstruction intervals, 5-mm thickness without a gap with a standard algorithm, and 1-mm thickness with 5-mm gaps with a high-frequency algorithm. Images were obtained after intravenous injection of 100 mL iopromide 300 (300mg I/mL Ultravist, Bayer Pharma, Berlin, Germany) at a rate of 2.5 mL/sec using a power injector, with a 50 second delay following contrast medium injection.

### Quantitative CT Analysis

Digital Imaging and Communications in Medicine data of the CT images were loaded to the ImageJ (Bethesda, Maryland; http://rsbweb.nih.gov/ij/) for lesion segmentation. The tumor regions of interest (ROIs) for analysis were drawn by two fellowship-trained academic radiologists with 1 year of chest radiology experience, and confirmed by an experienced radiologist with 20-years of experience in thoracic radiology. In the setting of the lung (width, 1500 Hounsfield unit [HU]; level, -700 HU) window, regions of interests in the tumors were semi-automatically selected by clicking on a part of the tumor and following automatic selection of the lesion by attenuation-based region growing method. Adjacent vessels, bronchus and chest wall in the ROIs were separated from the tumor manually by reviewing mediastinal (width, 450 HU; level 50HU) window setting. After a lesion was selected, quantitative histogram analyses were automatically calculated. Both 1-mm and 5-mm CT data were used to draw each region of interest, independently by the two radiologists, and agreement was reached between them on which representative axial image of the tumor to select for evaluation. If the regions of interest for the 1-mm and 5-mm images were different, an experienced chest radiologist decided which axial level of the images should be used for analysis. To compare the results of the 1-mm and 5-mm CT images, the regions of interest on the 1-mm and 5-mm CT images were juxtaposed, and one of them was re-delineated if they did not match.

Diameter, perimeter, area, attenuation, circularity, aspect ratio, roundness, skewness (symmetry of the pixel distribution), kurtosis (sharpness of the peak of the pixel distribution), and entropy (complexity of the pixel distribution) of tumors were obtained. Diameter was defined as the longest distance between any two points on the boundary of the ROI. Perimeter was defined as the length of the outside boundary of the ROI (mm), and area was defined as the area of the ROI in mm^2^. Attenuation was defined as the average HU within the ROI. Circularity was defined as $4\pi \times \frac{\left[Area\right]}{{\left[Perimeter\right]}^{2}}$ with a value of 1.0 indicating a perfect circle. Similarly, the aspect ratio was defined as the ratio of the width to the height of an ellipse fitted to the ROI $\frac{\left[Major\;Axis\right]}{\left[Minor\;Axis\right]}$. Roundness was defined as the ratio of the tumor area to the calculated circular area formed by the major axis: $4\times \frac{\left[Area\right]}{\pi \times {\left[Major\;Axis\right]}^{2}}$. As this value approaches 0.0, the tumor has an increasingly elongated shape. Skewness is defined as $E\left[{\left(\frac{X-\mu }{\sigma }\right)}^{3}\right]$, where X = attenuation, *σ* = mean of attenuation, *μ* = standard deviation of attenuation. Kurtosis is defined as $k=\frac{{E(x-\mu )}^{4}}{\sigma^{4}}-3$, where x = attenuation, *σ* = mean of attenuation, *μ* = standard deviation of attenuation. Entropy is defined as $\sum_{i=1}^{n}p(x_{i})\;\log\;p(x_{i})$, where, *x*_*i*_ = frequency from histogram of ROI, *p*(*x*_*i*_) = probability on histogram. The last three parameters are derived using the CT attenuation values (HU), and if the attenuation values of pixels are plotted, the three values could be derived automatically using ImageJ software.

### Statistical Analysis

Categorical variables are presented as numbers and percentages, and continuous variables were recorded as means and standard deviations or medians with interquartile ranges. Quantitative CT parameters obtained from the 1-mm and 5-mm data were compared using intraclass correlation coefficients (ICCs). The κ index was interpreted as follows: < 0.20, poor agreement; 0.21–0.40, fair agreement; 0.41–0.60, moderate agreement; 0.61–0.80, substantial agreement; and 0.81–1.00, excellent agreement. Univariate logistic analysis and multivariable logistic regression analysis with forward conditional method were used to analyze the independent prognostic factors of quantitative CT measurements on tumor recurrence using the 1-mm data. To assess the discriminatory power of the quantitative CT features, receiver operating characteristic curves were used. Cox regression analysis was also performed to compare the results from the logistic regression analysis. All statistical analyses were done with SPSS (version 21.0, SPSS Inc., Chicago, IL, USA).

## Results

### Patients’ Characteristics

Of the 194 patients, 81 were men. Tumor recurrence was noted in 25 patients, with local recurrence in 4 patients and distant metastasis in 21 patients. The median follow-up time from operation to recurrence was 1.9 years (range 0.5–2.6 years). General patient and tumor characteristics, including a comparison of the clinical and pathologic characteristics of the patients with and without tumor recurrence, are presented in Table 1. Clinical and CT findings of all patients are included in S1 File.

**Table 1:** Patient and Tumor Characteristics.

Variable	No recurrence (n = 169)		Recurrence (n = 25)			*P* value
Age, year	61.3 ± 9.0		61.6 ± 8.1			0.86
Male, n (%)	69 (40.8)		12 (48.0)			0.50
Smoking (no or ex-: current smoker)	52: 117		12: 13			0.09
Pack year	8.4 ± 17.3		10.2 ± 14.6			0.63
Lepidic pattern, %	24.1 ± 30.3		5.6 ± 10.3			<0.001
Lymphovascular invasion (+), n (%)							
	14 (8.3)		7 (28.0)			0.003
Visceral pleural invasion (+), n (%)	26 (15.4)		11 (44.0)			<0.001
Lymph node number, n	0.1 ± 0.7		0.2 ± 0.4			0.64
Peritumoral interstitial thickening (+), n (%)							
	5 (3.0)		4 (16.0)			0.02*
Pathologic diameter, mm	21.7 ± 10.7		31.5 ± 14.0			<0.001
Stage						0.01
Stage 1	156 (92.3)		19 (76.0)			
Stage 2	13 (7.7)		6 (24.0)			
Pathologic T stage, n						0.008^†^
T1a	84 (49.7)		4 (16.0)			
T1b	56 (33.1)		11 (44.0)			
T2a	25 (14.8)		7 (28.0)			
T2b	4 (2.4)		3 (12.0)			
Pathologic N stage						0.03*
N0	159 (94.1)		20 (80.0)			
N1	10 (5.9)		5 (20.0)			
EGFR (+), n (%)	124 (73.4)			22 (88.0)		0.14*	
Micropapillary or solid predominant (+), n (%)							
	3 (1.8)		0 (0.0)			1.00*
MaxSUV on PET/CT	4.0 ± 2.7		6.1 ± 3.6			0.009

Data are presented as mean ± standard deviation or n (%).

Student t-test and Pearson Chi square test (*Fisher's exact test) were used.

(+), positive; MaxSUV, maximum standardized uptake value; PET/CT, positron emission tomography/computed tomography.

^†^The P-value was obtained from the comparison between T1 and T2 stages using Pearson Chi square test.

### Comparison of Quantitative Parameters between 1-mm and 5-mm CT

The ICCs between the quantitative CT parameters obtained from the 1-mm and 5-mm data showed excellent agreement in terms of diameter, perimeter, area, mean attenuation, aspect ratio and entropy (Table 2). Circularity and roundness showed substantial agreement. However, skewness and kurtosis presented moderate agreement.

**Table 2:** Intraclass correlation coefficients (ICC) between 1-mm and 5-mm thickness CT data.

Variable	ICC	95% CI		*P* value
Diameter, mm	0.98	0.97	0.99	<0.001
Perimeter, mm	0.95	0.93	0.96	<0.001
Area, mm^2^	0.99	0.98	0.99	<0.001
Mean attenuation, HU	0.96	0.95	0.97	<0.001
Circularity	0.71	0.62	0.79	<0.001
Aspect ratio	0.81	0.75	0.86	<0.001
Roundness	0.80	0.74	0.85	<0.001
Skewness	0.60	0.47	0.70	<0.001
Kurtosis	0.53	0.37	0.64	<0.001
Entropy	0.85	0.80	0.89	<0.001

CI, confidence interval; HU, Hounsfield unit.

### Quantitative CT Parameters and Tumor Recurrence

**Fig 2:**
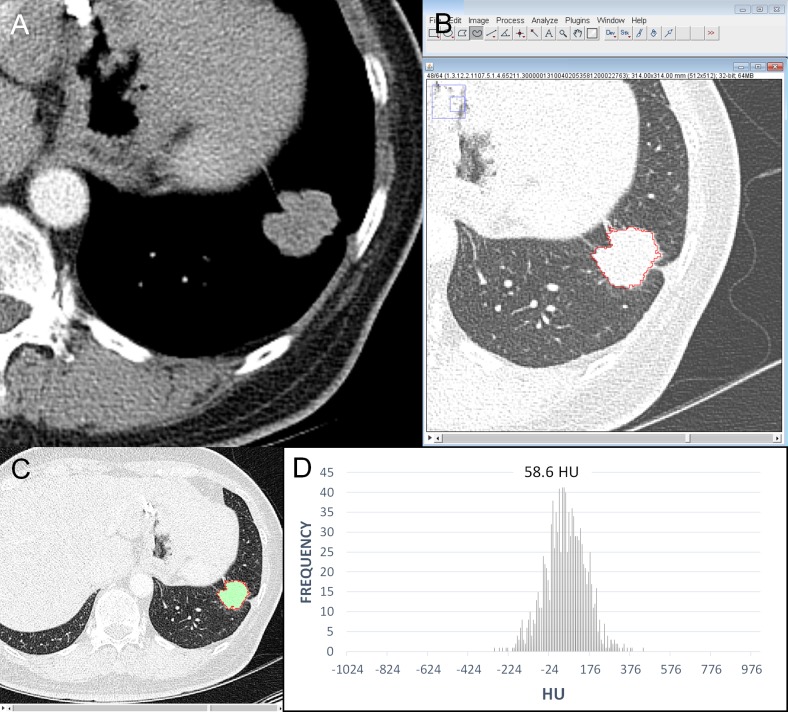
An example of quantitative CT analysis in a recurred adenocarcinoma. (A) CT scan and (B) screenshot of quantitative analysis show a 2.8-cm irregular nodule in the left lower lobe. The TNM stage was T1bN0M0 with stage IA. (C) The graph shows CT number (Hounsfield unit) distribution of the tumor. The perimeter of the tumor was 95.50 mm; area of tumor was 409.96 mm^2^; mean attenuation, 58.61 HU (Hounsfield unit); circularity, 0.57; aspect ratio 1.15; roundness, 0.87; skewness, -0.018; kurtosis, 0.39; and entropy, 7.01.

**Fig 3:**
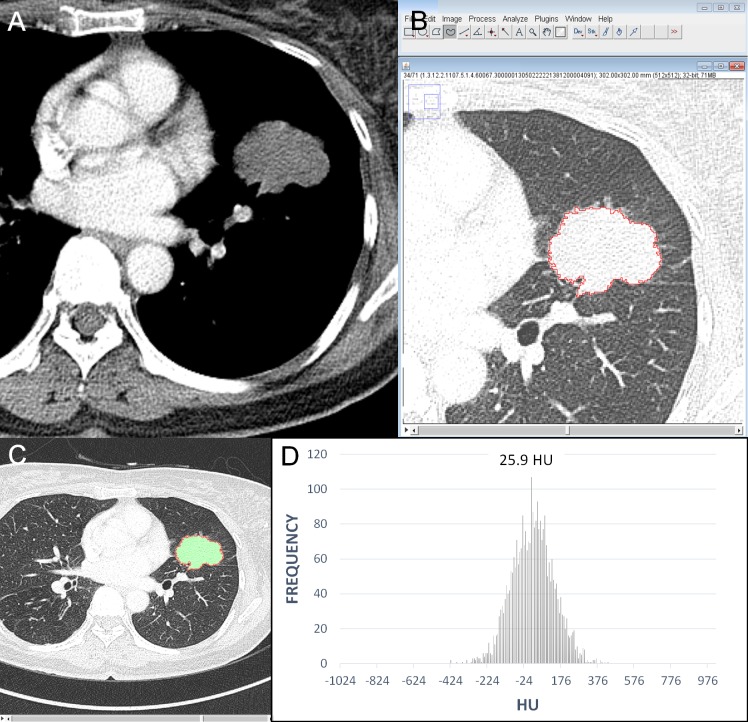
An example of quantitative CT analysis in an adenocarcinoma without recurrence. (A) CT scan and (B) screenshot of quantitative analysis show a 4.6-cm irregular nodule in the left upper lobe (T2aN1M0, stage IIA). (C) The graph shows CT number (Hounsfield unit) distribution of the tumor. The perimeter of the tumor was 159.02 mm; area of tumor was 1020.79 mm^2^; mean attenuation, 25.91 HU (Hounsfield unit); circularity, 0.51; aspect ratio 1.48; roundness, 0.67; skewness, 0.014; kurtosis, 0.21; and entropy, 7.06. The tumor attenuation is low, and is expected to no recurrence and the patient alive without recur for two years.

**Fig 4:**
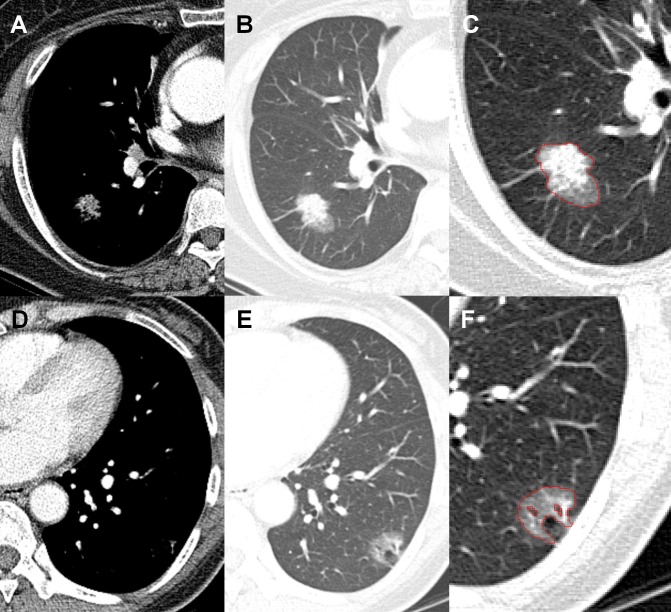
Part-solid adenocarcinomas with or without recurrence. (A) Mediastinal setting and (B) lung setting of CT images show a 3.2-cm part-solid adenocarcinoma in the right lower lobe (T2aN0M0, stage Ib). The lesion shows -315.27 HU (Hounsfield unit) and the area of lesion was 443.95 mm^2^. (C) The region of interest (ROI) for the tumor drawn in the ImageJ was noted. Lung metastasis was developed two years after the surgery. (D, E) A 2.1-cm part-solid nodule in the left lower lobe. The tumor area was 247.65 mm^2^ and the attenuation was -445.93 HU. (F) Air-bronchogram within the lesion was excluded for the measured ROI of the lesion. There was no recurrence in this patient for recent three years.

The results of the univariate logistic regression analysis of the 1-mm and 5-mm thickness CT data are shown in Table 3. The diameter (odds ratio [OR], 2.26 for each 1-cm increase, *P* < 0.001), perimeter (OR, 1.18 for each 1-cm increase, *P* < 0.001), and area (OR, 1.35 for each 1-cm^2^ increase, *P* < 0.001) of tumors measured at 1-mm thickness CT data were associated with tumor recurrence. In the 5-mm thickness data, the parameters also showed statistically significant. The mean attenuation of the tumors was significantly correlated with tumor recurrence in both 1-mm and 5-mm thickness images (OR, 1.11 for each 10-HU increase, *P* <0.05) (Figs 2, 3 and 4). Circularity, aspect ratio, roundness of tumors and entropy were not significantly correlated with recurrence. Skewness and kurtosis were significantly associated with tumor recurrence only in the 5-mm thickness CT images obtained using a standard algorithm.

**Table 3:** Univariate logistic regression analysis of 1-mm and 5-mm thickness CT data.

Variable	No recurrence	Recurrence	OR	95% CI		*P* value
1-mm thickness CT						
Diameter, mm	23.67 ± 10.17	35.19 ± 13.13	1.09	1.05	1.13	<0.001
Perimeter, mm	80.49 ± 40.61	119.88 ± 56.74	1.02	1.01	1.03	<0.001
Area, mm^2^	245.82 ± 240.12	564.51 ± 405.23	1.003	1.001	1.004	<0.001
Mean attenuation, HU	-176.13 ± 229.12	-4.33 ± 116.0	1.01	1.002	1.01	0.002
Circularity	0.47 ± 0.15	0.51 ± 0.17	5.85	0.39	88.87	0.20
Aspect ratio	1.46 ± 0.34	1.52 ± 0.35	1.60	0.52	4.97	0.42
Roundness	0.72 ± 0.14	0.69 ± 0.14	0.24	0.01	4.56	0.34
Skewness	-0.09 ± 0.87	-0.28 ± 1.06	0.77	0.46	1.28	0.31
Kurtosis	1.08 ± 3.29	2.18 ± 4.99	1.06	0.97	1.16	0.18
Entropy	6.78 ± 0.45	6.73 ± 0.47	0.84	0.34	2.07	0.70
5-mm thickness CT						
Diameter, mm	23.32 ± 9.94	33.09 ± 12.31	1.08	1.04	1.12	<0.001
Perimeter, mm	72.09 ± 33.51	102.54 ± 42.47	1.02	1.01	1.03	<0.001
Area, mm^2^	263.35 ± 248.47	530.73 ± 381.99	1.002	1.001	1.004	<0.001
Mean attenuation, HU	-238.25 ± 221.30	-33.52 ± 122.58	1.01	1.003	1.01	<0.001
Circularity	0.59 ± 0.16	0.61 ± 0.15	2.17	0.15	32.22	0.57
Aspect ratio	1.45 ± 0.35	1.52 ± 0.39	1.64	0.56	4.75	0.37
Roundness	0.72 ± 0.14	0.70 ± 0.15	0.26	0.01	4.84	0.36
Skewness	-0.22 ± 0.93	-0.94 ± 1.09	0.50	0.33	0.76	0.001
Kurtosis	0.45 ± 2.53	2.81 ± 5.23	1.18	1.05	1.32	0.005
Entropy	6.92 ± 0.50	6.72 ± 0.52	0.51	0.24	1.07	0.07

CI, confidence interval; HU, Hounsfield unit; OR, odds ratio.

**Fig 5:**
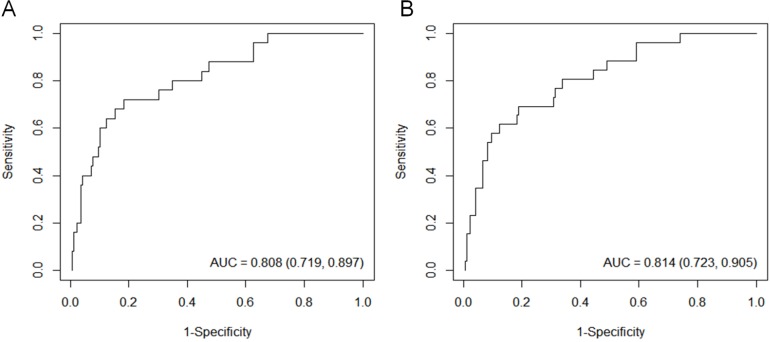
**Receiver operating curves to demonstrate the diagnostic performance of the independent predictive factors for tumor recurrence in both (A) 1-mm and (B) 5-mm CT data.** The overall C-indices for 1-mm and 5-mm thickness CT images using two parameters (area and mean attenuation of tumors) are 0.80 (95% CI, 0.72–0.90) and 0.81 (0.72–0.90), respectively. AUC, area under the curve (C-index); CI, confidence interval.

In a multivariable analysis of the 1-mm CT data, area (OR, 1.22 for each 1-cm^2^ increase; *P* = 0.003) and mean attenuation (OR, 1.05 for each 10-HU increase; *P* = 0.022) were independently associated with tumor recurrence (Table 4). For each 1-cm^2^ increase in tumor size, the odds ratio increased 1.24-fold, while in terms of the mean attenuation, the odds ratio increased by 1.05 times for each 10-HU increase. The results from the Cox regression analysis were similar to that of logistic regression methods, and the hazard ratios of the prognostic factors are presented as S1 Table. The receiver operating curves using the two independent predictive factors had a high diagnostic performance in both the 1-mm and 5-mm data (C-indices = 0.80 and 0.81, respectively) (Fig 5).

**Table 4:** Univariate and multivariable logistic regression of the quantitative CT parameters measured on 1-mm and 5-mm thickness CT data.

Variable	Univariate				Multivariable			
	OR	95% CI		*P* value	OR	95% CI		*P* value
1-mm thickness CT								
Diameter, mm	1.08	1.04	1.12	<0.001				
Perimeter, mm	1.02	1.01	1.03	<0.001				
Area, mm^2^	1.003	1.001	1.004	<0.001	1.002	1.001	1.004	0.003
Mean attenuation, HU	1.01	1.00	1.01	0.002	1.005	1.001	1.009	0.022
5-mm thickness CT								
Diameter, mm	1.08	1.04	1.12	<0.001				
Perimeter, mm	1.02	1.01	1.03	<0.001				
Area, mm^2^	1.002	1.001	1.004	<0.001	1.001	1.001	1.003	0.048
Mean attenuation, HU	1.01	1.00	1.01	<0.001	1.006	1.002	1.01	0.003
Skewness	0.50	0.33	0.76	0.001				
Kurtosis	1.18	1.05	1.32	0.005				

CI, confidence interval; HU, Hounsfield unit; OR, odds ratio.

## Discussion

Low dose CT has become increasingly popular for lung screening [21]. As a consequence, early detection of adenocarcinoma before lymph node metastasis during medical checkups is not uncommon, especially in high risk patients. The spectrum of adenocarcinoma of the lung is wide and varies from sub-solid nodules (frequently seen in atypical adenomatous hyperplasia, adenocarcinoma in situ) or part-solid nodules (frequently seen in minimally invasive adenocarcinoma) to obviously solid nodules. Visual assessment of opacity is not always easy, and inter-reader or intra-reader agreement is not very good even when expert chest radiologists are involved [22]. Moreover, the development of a systematic method for differentiating recurrence from non- recurrence of adenocarcinoma of the lung is important given that there is concern regarding the use of adjuvant therapy versus watchful follow-up after surgical resection.

To provide a more precise analysis of the characteristics of lung cancer and the expected outcomes including recurrence, a more detailed and objective assessment of lung cancer using preoperative CT is needed. CT texture analysis has been suggested as a potential tool for predicting clinical outcomes in a variety of cancers [10–14, 16, 23, 24]. Some investigators have adopted computerized texture analysis to evaluate resectable lung nodules [18, 25]. Chae et al. found that the mass and kurtosis differentiated between preinvasive lesions and invasive pulmonary adenocarcinomas that appeared as part-solid ground-glass nodules (0.625–1.25 mm slice thickness) [25]. However, prediction of recurrence using preoperative CT in stage I, II lung adenocarcinoma after curative resection using visual assessment is not easy because of substantial overlap between the CT imaging features. We have therefore attempted to discriminate predicted recurrence from non-recurrence using quantitative CT features. Multivariable analysis showed that area and mean attenuation were independent predictors of tumor recurrence. These two independent factors performed excellently in differentiating recurrence from non- recurrence with a C-index value of 0.81in both 1-mm and 5-mm data.

A previous study reported that entropy was an independent prognostic factor for survival in both curative and palliative patients with non-small cell lung cancer [16]. Ahn et al. reported that entropy, skewness, and mean attenuation (5-mm slice thickness) were significantly associated with overall survival in patients with advanced non-small cell lung cancer treated with definitive concomitant chemotherapy [17]. However, in the present study, entropy showed no significant correlation with tumor recurrence in surgically resectable patients. In terms of skewness, because the correlation between the values obtained from the 1-mm and 5-mm CT data was poor, the choice of slice thickness and different kernels might be considerable factors to affect the study results. In our work, skewness and kurtosis were correlated with tumor recurrence only in the 5-mm thickness CT data obtained using a standard kernel. Diameter, perimeter, area and mean attenuation were correlated with tumor recurrent in both the 1-mm and 5-mm CT data. Those parameters are promising as CT imaging biomarkers for predicting recurrence of surgically-resected lung adenocarcinoma.

In this work, we compared 1-and 5-mm reconstructions, which are widely accepted CT protocols in routine practice. The ICC values for diameter, perimeter, area, and entropy were excellent. However, the ICCs for circularity, aspect ratio, and roundness were moderate. These findings were expected because 1-mm thickness CT images are noisy but sharper than 5-mm images, and the tumor margins drawn tend to be much more irregular. In the same way, the fact that skewness and kurtosis showed moderate agreement between 1-mm and 5-mm data, this suggests the importance of image thickness and reconstruction kernel selection for quantitative CT analysis. Quantitative CT measurements for assessing tumor heterogeneity are highly dependent not only on image quality, but also on the thickness or coarseness of the images [15]. Further studies of the use of different image thicknesses for assessing quantitative parameters could be helpful in standardizing the CT protocol.

Maximum standardized uptake value is also a significant preoperative predictor for surgical outcomes of lung adenocarcinoma [26, 27]. In this study, the mean maxSUV values in both patients with and without recurrence were 6.2 and 4.3, respectively. This result is well correlated with the findings of a previous report that solid adenocarcinomas with maxSUV values of ≥4.4 was significantly associated with tumor invasiveness [25]. In that study, the ratio of the maximum diameter of consolidation to the maximum tumor diameter on CT ≥53% was also a significant factor relate to the tumor invasiveness for subsolid adenocarcinoma. We did not measure the consolidative areas and ground-glass opacities of tumors separately. However, considering that subsolid adenocarcinoma with ground glass opacities are presented as low attenuation compared to the solid components of tumors, the finding that mean attenuation of tumor on CT is associated with tumor recurrence could be explained.

This study had several limitations. First, it is a retrospective study in a single referral center, which means that it is subject to selection bias. To reduce bias, we included consecutive patients who performed preoperative CT using a dedicated scanner over a period of one year obtained from our lung cancer registry. Second, because this retrospective study used CT scans which are previously obtained in routine practice, we could not directly compare the effect of slice thickness or kernels alone. Although it is difficult to interpret the impact of slice thicknesses or different kernels alone, the choice of slice thickness and reconstruction kernels might be considerable factors to affect the quantitative analysis results. Future studies to analyze different slice thickness CT images based on the same kernel, or different reconstruction kernels using the same slice thickness may be needed to allow direct comparison. Third, although we used in-house software based on plug-in package for ImageJ, which is easily available on the internet, the quantitative features were derived by semi-automatic segmentation. Moreover, tumor ROI was defined on a 2D slice rather than 3D volume measurements. However, 2D tumor segmentation could be performed within a few minutes by the method of attenuation difference-based semiautomatic selection, and might be easily applicable in the clinical setting. Finally, we did not include lung cancer other than adenocarcinoma in this study. Moreover, there was no validation group to assess the performance of our model, and the recurrence group was small number compared to the large number of potential predictors. Thus, the generalizability of these results to other users is unknown, and further studies to validate the present results would be of value.

## Conclusions

In conclusion, independent tumor area and mean attenuation are significant differentiators of tumor recurrence in patients with surgically resected adenocarcinoma of the lung. Other quantitative parameters did not show significant correlations with the tumor recurrence. Evaluation of the well-focused CT morphologic features could provide more accurate tumor assessment.

## Supporting Information

S1 Table Univariate Cox regression analysis of the quantitative CT parameters. (DOCX)

S1 File CT findings and clinical features of all patients. (XLSX)
