# Supplementary material for: Quantitative Computed Tomography Features for Predicting Tumor Recurrence in Patients with Surgically Resected Adenocarcinoma of the Lung
Source: PLoS One. 2017 Jan 9;12(1):e0167955. doi: 10.1371/journal.pone.0167955 (PMC5221878; doi:10.1371/journal.pone.0167955)
Supplement: S1 Table — (DOCX) [file pone.0167955.s001.docx]

**S1 Table. Univariate Cox regression analysis of the quantitative CT parameters**

| Variable | HR | 95% CI | | P-value |
| --- | --- | --- | --- | --- |
| 1-mm thickness CT | | | | |
| Diameter | 1.08 | 1.05 | 1.11 | <0.001 |
| Perimeter | 1.02 | 1.01 | 1.02 | <0.001 |
| Area | 1.002 | 1.001 | 1.003 | <0.001 |
| Mean attenuation | 1.01 | 1.002 | 1.01 | 0.002 |
| Circularity | 5.84 | 0.47 | 72.01 | 0.17 |
| Skewness | 0.77 | 0.48 | 1.22 | 0.26 |
| Kurtosis | 1.04 | 0.97 | 1.11 | 0.28 |
| Aspect ratio | 1.55 | 0.57 | 4.21 | 0.18 |
| Roundness | 0.23 | 0.02 | 3.26 | 0.28 |
| Entropy | 1.06 | 0.46 | 2.44 | 0.90 |
| 5-mm thickness CT | | | | |
| Diameter | 1.08 | 1.05 | 1.12 | <0.001 |
| Perimeter | 1.02 | 1.01 | 1.03 | <0.001 |
| Area | 1.002 | 1.001 | 1.003 | <0.001 |
| Mean attenuation | 1.01 | 1.003 | 1.01 | <0.001 |
| Circularity | 2.46 | 0.20 | 29.99 | 0.48 |
| Skewness | 0.60 | 0.43 | 0.83 | 0.002 |
| Kurtosis | 1.10 | 1.03 | 1.18 | 0.004 |
| Aspect ratio | 1.45 | 0.57 | 3.71 | 0.44 |
| Roundness | 0.32 | 0.02 | 4.69 | 0.41 |
| Entropy | 0.60 | 0.29 | 1.21 | 0.15 |
